# Credit risk assessment of small and micro enterprise based on machine learning

**DOI:** 10.1016/j.heliyon.2024.e27096

**Published:** 2024-03-07

**Authors:** Zhouyi Gu, Jiayan Lv, Bingya Wu, Zhihui Hu, Xinwei Yu

**Affiliations:** a School of Information Technology, Zhejiang Financial College, Hangzhou 310018, China; b Library of Huzhou University, Huzhou 313000, China; c School of Marine Engineering Equipment, Zhejiang Ocean University, Zhoushan 316022, China

**Keywords:** Small and micro enterprises, Imbalanced processing, Machine learning, Scorecard model

## Abstract

Small and micro enterprises are pivotal in national economic and social development. To foster their growth, managing their credit risks scientifically is crucial. This study starts by examining the credit information of these enterprises. We use imbalanced sample processing algorithms to ensure a balanced representation of minority-class samples. Then, a machine learning classifier is employed to identify key factors contributing to these enterprises' low credibility. Based on these factors, an XGBoost scoring card model is developed. The study reveals: firstly, the integration of the SMOTE algorithm with the XGBoost model exhibits certain performance advantages in handling imbalanced datasets; secondly, trustworthy financial information remains at the heart of crucial risk determinants; thirdly, the XGBoost scoring card model based on significant features effectively enhances the accuracy of credit risk assessment. These insights provide both theoretical references and practical tools for enhancing the robustness of small and micro enterprises, facilitating early warnings on credit risks, and refining financing efficiency.

## 1 Introduction

Small and micro enterprises are critical drivers of economic progress and job creation globally. According to the World Bank, these businesses constitute nearly 90% of all enterprises worldwide, accounting for over half of global employment.^1^ China's small and micro enterprise sector has seen unprecedented growth in the last two decades. Today, these enterprises make up 95.80% of all businesses in the country.^2^ Nevertheless, despite their significant impact on China's economic landscape and societal framework, these enterprises encounter substantial obstacles when seeking financial support, especially from commercial banks. Such financing challenges consistently impede their growth potential. A clear relationship emerges between these financing difficulties, elevated financing costs, and the credit risk assessments targeted at them. As the perceived credit default risk for a small or micro enterprise decreases, the financing costs they incur also tend to drop, broadening their avenues for securing funding [1].

The rise of the digital era further enriches this context. In contrast to traditional financial risk strategies, which predominantly rely on financial records, the advent of big data has expanded credit assessments for small and micro enterprises by introducing a diverse set of credit indicators [2]. This results in a broader scope of information available for scrutiny. However, this wealth of information carries its own set of challenges. Not all credit data points have equal significance in risk assessments. Thus, sifting through the paramount factors amid many potential contributors and constructing a solid statistical model based on these key elements stands at the heart of this research.

The domain of credit risk assessment for small and micro enterprises has mainly been bifurcated into two principal strands of research [3]. The first strand revolves around the types of information harnessed for these assessments, while the second dives into the methodologies and techniques employed. Informational foundations for assessment can be succinctly stratified into four primary dimensions: financial information, micro-level enterprise behavior information, public credit information, and information obtained from third-party sources. (1) Financial information: Traditionally, credit risk assessment has been deeply entrenched in financial metrics. Early trailblazers in this realm, such as Beaver and Altman, employed a multitude of financial indicators to forecast potential corporate failures [4,5]. [6] highlights that financial ratios are vital for assessing corporate financial health, with the most common ratios in prediction models being the current ratio, total liabilities to total assets ratio, and total sales to total assets ratio [7]. broaden the scope, leveraging an array of financial data, from the current ratio and total asset turnover rate to other pivotal financial ratios. Taking a more holistic approach [8]. introduces federated learning as a novel solution to tackle the issue of information asymmetry between banks and enterprises in credit assessments [9]. underscore the profound influence of liquidity and profitability on credit risk, emphasizing the magnified susceptibility of small and micro enterprises to debt-induced credit risk. Nevertheless, the financial data landscape for these enterprises is often marred by gaps and a lack of transparency, rendering an overreliance on such data potentially misleading [10,11]. [[12], [13], [14]] indicate that the field of credit risk assessment is evolving towards a more diverse and sophisticated technological approach, encompassing non-financial indicators, artificial intelligence, and fuzzy logic methods. (2) Micro-level enterprise behavior information: Inspired by enterprise behavior theory, emerging methodologies aim to address the limitations inherent in financial data. For instance Ref. [15], proposes a “two-dimensional” credit evaluation paradigm, blending traditional financial metrics with micro-behavioral insights. Pushing the envelope further [16], introduces DeepRisk, amalgamating demographic and financial behavioral data [17]. craft a specialized risk assessment system for e-commerce-based small and micro enterprises, focusing on operational behavior and service provision nuances. [18], Similarly, validate the enhanced predictive prowess by embedding insights from management dynamics and employee metrics. (3) Public credit information: The proliferation of computer technology has augmented the accessibility and convenience of information, leading to an upsurge in the utilization of public credit information in research [19]. Advocates of this approach, like [20], argue its supremacy in curtailing the pitfalls of information asymmetry. This method offers a more holistic, reliable, and cost-effective lens than solely using financial information. Echoing this sentiment [21], illustrates the potent influence of public credit releases by governmental agencies on enterprise credit ratings. (4) Information obtained from third-party sources: Beyond the conventional reservoirs of information, some scholars are championing the incorporation of third-party data sources into the assessment matrix, arguing its potential to enrich the credit evaluation ecosystem. For example [22], utilizes big data from social media to predict corporate credit ratings, highlighting that with the proliferation of the internet and social media, a company's appeal on social platforms has become an alternative means for financial institutions to determine credit ratings [23]. proposes a blockchain-based decentralized model, reducing reliance on traditional data sources. In conclusion, research on credit risk assessment for small and micro enterprises is evolving, expanding, and becoming more sophisticated. While foundational financial information remains seminal, the inclusion of behavioral data, public credit records, and third-party insights is reshaping the landscape, promising more holistic and accurate assessments.

Research into credit risk assessment methods for small and micro enterprises predominantly falls into three methodological buckets: expert analysis, statistical techniques, and machine learning [11,24]. Expert analysis: This approach profoundly relies on an assessor's expertise and professional judgment. It involves deducing the creditworthiness of enterprises based on the assessor's experience. Due to its inherent subjectivity, evaluations for the same entity can substantially diverge based on the appraiser. This variability challenges the quest for assessment impartiality [11,[25], [26], [27]]. Statistical techniques: To transcend the subjective limitations of expert analysis, many scholars pivot to statistical methods. Discriminant analysis, logistic regression, and probit models rank among the most utilized techniques [28]. Early implementations include Fisher's discriminant analysis model [29] and Altman's Z-score model [5]. The advent of logistic regression in predicting defaults is introduced by Ref. [30]. Comparatively, logistic models have an edge over discriminant analysis in predictive accuracy [31], primarily due to their less constraining model assumptions [32]. Integrating correlation analysis with Probit regression [33], adeptly differentiates between defaulting and non-defaulting customers. Empirical tests conducted by Ref. [34] using a unique dataset of French SMEs from 2012 to 2018 suggest the superior accuracy of Probit models over logistic models. However, the linear constraints of evaluation indicators and the assumptions about the normal distribution of indicators are challenges inherent in these statistical methods, limiting their efficacy in corporate credit risk assessment [35]. Machine learning: With the digital revolution at the cusp of the 21st century, credit evaluations witnessed a paradigm shift towards intelligence-driven methods. Drawing inspiration from the application and research of machine learning in engineering [[36], [37], [38]], some scholars have started introducing these methodologies into credit assessment for small and micro enterprises. This cross-disciplinary approach signifies a pioneering step in leveraging advanced analytical tools to enhance the accuracy and efficiency of credit assessments in the business sector [[39], [40], [41]]. [42,43] delves into utilizing Support Vector Machines (SVM) and Neural Networks [44], showcases their efficacy in complex financial environments. Concurrently [45], merges SVM with Logistic Regression, highlighting significant advancements in predictive accuracy for SME credit decisions [46]. applies the Random Forest algorithm for micro-enterprises credit risk modeling, emphasizing its robustness in accuracy and interpretability [47]. integrates the XGBoost model into enterprise credit risk assessments, achieving enhanced precision in audit opinion predictions, marking a significant stride in the domain. Subsequently, an advanced XGBoost algorithm is proposed by Ref. [48], demonstrating its supremacy in prediction accuracy, notably outpacing traditional methodologies like discriminant analysis and logistic regression. With the deepening application of machine learning models, a growing body of scholarly work is turning attention towards integrative research that combines these models with cutting-edge technologies such as the Internet of Things (IoT) and cloud computing [49,50]. This trend benefits innovative research in credit assessment for small and micro enterprises.

Current research has made considerable strides in credit risk assessment for small and micro enterprises. However, there remain opportunities for deeper exploration. One pressing need is the refined usage of available informational components to portray these enterprises' more intricate credit landscape. Existing literature has drawn from corporate financial information [[4], [5], [6], [7], [8], [9]], micro-level enterprise behavior information [[15], [16], [17], [18]], public credit information [[19], [20], [21]], and information obtained from third-party sources [22,23] for crafting assessment frameworks. However, there is a lack of comprehensive consideration of these information elements. Additionally, the continuous drive to bolster the precision of assessment models remains salient. Previous researches [[24], [25], [26], [27], [28], [29], [30], [31], [32], [33], [34], [35], [36], [37], [38], [39], [40], [41], [42], [43], [44], [45], [46], [47], [48], [49], [50]] have crafted predictive models through various methodologies, including expert analysis, statistical techniques, and machine learning. Even though these efforts have achieved commendable success in predictive fidelity, there is ample room for further enhancements and pioneering breakthroughs in the field.

**Fig. 1:**
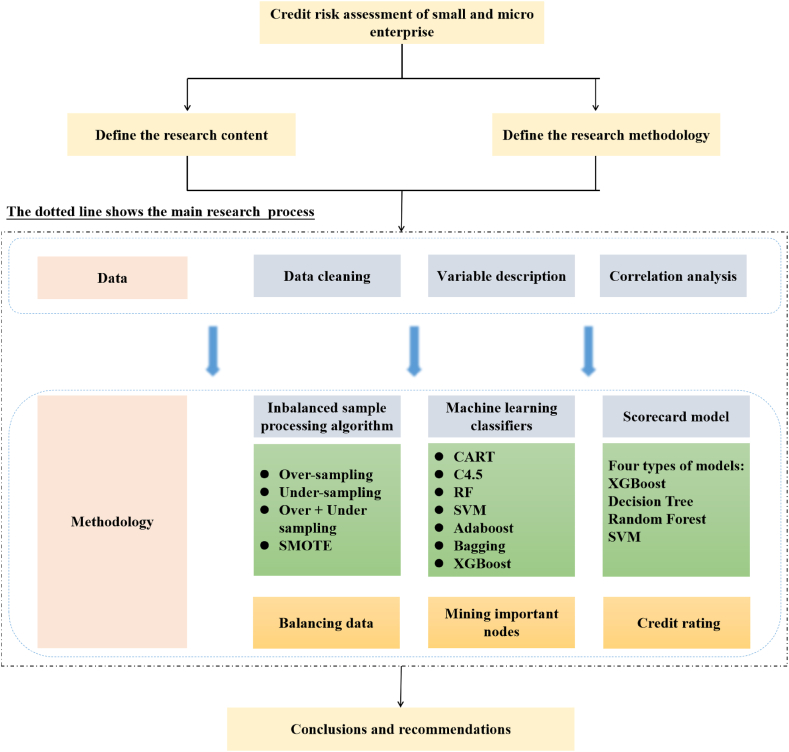
Research frameworks.

Addressing the previously noted challenges, this study evaluates the current dynamics and characteristics of small and micro enterprises. Beginning with financial information, enterprise micro-behavioral information, public credit information, and third-party access to information, we curate 811 valid datasets, each encompassing 20 distinct informational elements. Methodologically, the research crafts an integrated framework that synergizes imbalanced data rectification, strategic feature emphasis, and state-of-the-art machine learning methods. By reducing the dimensionality of data and establishing a tailored machine learning model, we not only elevate predictive precision but also quantify the creditworthiness of small and micro enterprises through a credit score (see Fig. 1). The potential innovations of this study are as follows: (1) Dataset Diversity: Our dataset stands apart in its uniqueness, encompassing financial information, enterprise micro-behavioral information, public credit information, and third-party access to information. This comprehensive dataset facilitates a more nuanced assessment of credit risks associated with small and micro enterprises. In future research endeavors, it is imperative to focus on the diverse information dimensions of small and micro enterprises, assessing their credit risk from multiple perspectives. (2) Innovative Methodology: By harnessing imbalanced sample processing algorithms, we address the challenges of underrepresented minority class samples. Furthermore, through advanced machine learning classifiers, we pinpoint crucial determinants impacting the diminished credibility of small and micro enterprises. Culminating our approach, we design an XGBoost scoring card model rooted in these paramount characterizing factors. This model strategy offers a novel perspective for future research, particularly in addressing high-dimensional data analysis challenges. Employing such a combined model approach facilitates dimensionality reduction and simplifies the complexity of parameter estimation. (3) Practical Relevance: Our findings offer significant real-world applications beyond theoretical contributions. They serve as a valuable resource for entities vested in fostering the robust growth of small and micro enterprises, enhancing credit risk forewarning mechanisms, and optimizing financing efficiency. Additionally, subsequent research could focus on model innovation based on important feature identification.

The structure of the paper unfolds across four segments: The initial section serves as the introduction, setting the context. Following this, the second segment details the data sources and correlation analysis. In the third section, we examine the empirical findings in depth. The final segment wraps up the study with a conclusion.

## 2 Data sources and analysis

### 2.1 Data sources and description of variables

The sample dataset utilized in this study originates from a third-party credit assessment company. It primarily encompasses financial information, micro-level enterprise behavior data, public credit data, and information obtained from third-party sources. Initially, the dataset contains records of 850 small and micro enterprises. However, after an exhaustive data cleansing process—addressing outliers and removing invalid entries—we are left with a refined enterprise directory of 811 entries.

The dataset comprises 20 variables. The “Total_score” stands as the dependent variable, representing the creditworthiness of small and micro enterprises. The remaining 19 are independent variables, offering insights into the fundamental characteristics of these enterprises, as outlined in Table 1.

**Table 1:** Overview and description of variables.

Serial Number	Variable Symbol	Meaning of Variables
1	GM01	Last Year's Total Assets
2	GM02	Registered Capital
3	GM03	Last Year's Total Profit
4	GM04	Last Year's Operating Revenue
5	ZX01	Return on Equity (ROE) of Last Year
6	ZX02	Return on Assets (ROA) of Last Year
7	ZX03	Asset-Liability Ratio of Last Year
8	ZX04	Owner's Equity of Last Year
9	ZX05	Years in Operation
10	ZX06	Year-on-Year Index of Water, Electricity, and Gas Consumption
11	ZX07	Number of Intellectual Property Rights
12	GL01	Legal Representative's Contribution
13	GL02	Executive's Contribution
14	GL03	Shareholder Strength Score
15	GL04	Legal Representative's Age
16	GX01	Number of Social Security Contributions/Number of Employees
17	GX02	Total Annual Tax Payment
18	HG01	Compliance Performance
19	RY01	Bonus Points for Honors
20	Total_score	Creditworthiness: High = 1, Low = 0.

### 2.2 Correlation analysis

**Fig. 2:**
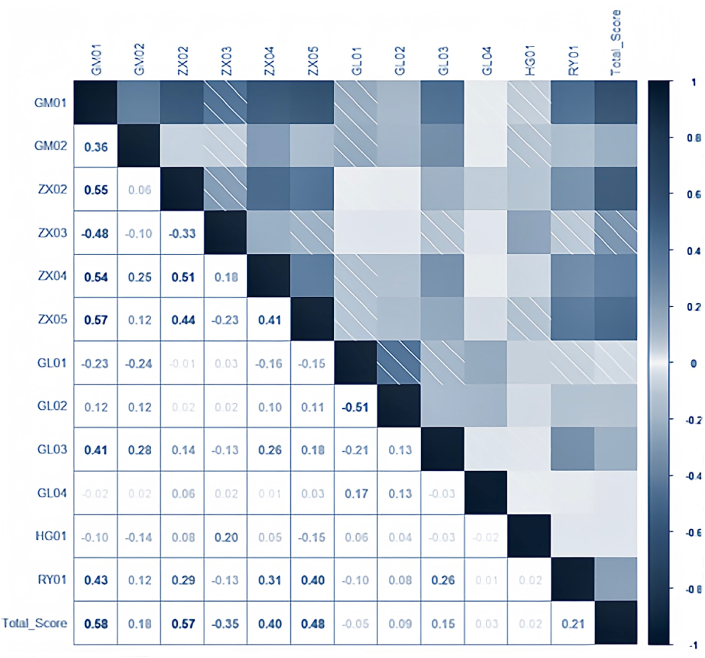
Correlation coefficient matrix heatmap between variables.

We employ correlation analysis to explore the relationships between variables. Fig. 2 provides a heatmap of the correlation coefficient matrix, spotlighting the 12 explanatory variables with the strongest correlations to the target variable. The heatmap reveals that most correlations between explanatory and target variables are notably weak. Notably, most correlation coefficients between these variables have absolute values not exceeding 20%, suggesting that the dataset might have limitations when utilized in traditional models. Additionally, the low correlation coefficients among the explanatory variables suggest minimal multicollinearity.

## 3 Credit risk assessment of small and micro enterprises based on imbalanced samples

### 3.1 Handling imbalanced samples

In this study, we have collected 811 valid data samples. Among these, 84 enterprises exhibit poor creditworthiness, making up only 10.36% of the samples. Conversely, 727 enterprises demonstrate good creditworthiness, comprising 89.64% of the samples. This pronounced disparity indicates an imbalanced dataset. In typical data analysis, the dominant category can overshadow the minority, potentially skewing the model's results. Such skewness can compromise the effectiveness of the assessment. As a result, rectifying this data imbalance is crucial.

“Over-sampling” seeks to balance the dataset by replicating instances from the minority class, ensuring the original data remains unchanged. Conversely, “Under-sampling” achieves balance by randomly discarding samples from the majority class. The SMOTE algorithm takes a unique approach: It reviews the minority class instances and synthesizes new samples using interpolation, thus equalizing the dataset's distribution. We allocate 75% of the primary data for this research as our training dataset. Subsequently, we apply over-sampling, under-sampling, a blend of both, and SMOTE to address data imbalance in the training set [51]. Table 2 showcases the results.

**Table 2:** Sampling techniques for imbalanced data.

Sampling Method	Dataset	Poor Creditworthiness	Good Creditworthiness	Total
	Raw Data	84（10.36%）	727（89.64%）	811
Simple random sampling	Test Set	21（10.34%）	182（89.66%）	203
	Training Set	63（10.36%）	545（89.64%）	608
Over-sampling	New Training Set 1	545（50%）	545（50%）	1090
Under-sampling	New Training Set 2	63（50%）	63（50%）	126
Over + Under sampling	New Training Set 3	309（50%）	309（50%）	608
SMOTE	New Training Set 4	378（44.47%）	472（55.53%）	850

### 3.2 Performance analysis

Classification models are pivotal in data analytics and can be broadly categorized into single classification methods and ensemble techniques. The former encompasses individual classifiers like decision trees, neural networks, and support vector machines, while the latter integrates techniques such as Bagging and Boosting. For our research, we have chosen seven algorithms spanning both categories. Our response variable is ‘creditworthiness,’ with possible values of 0 or 1. Using the imbalanced datasets from Table 2, we have implemented seven classifiers: CART, C4.5, RF, SVM, Adaboost, Bagging, and XGBoost. To gauge the performance of each classifier, we rely on six pivotal metrics: true positive rate, true negative rate, sensitivity, specificity, accuracy, and AUC.

Classifiers can produce varying outcomes even when using the same imbalanced data processing method. As depicted in Table 3, RF, AdaBoost, and XGBoost emerge superior across diverse metrics after applying a consistent imbalance data treatment. SVM ranks closely behind, while CART and C4.5 lag in their classification outcomes.

**Table 3:** Results of different classifiers.

	Evaluation Metrics	CART	C4.5	RF	SVM	AdaBoost	Bagging	XGBoost
Over-sampling	Sensitivity	0.920	0.940	0.990	0.970	0.990	0.980	0.960
	Specificity	0.950	0.940	0.810	1.000	0.810	0.760	0.970
	True Positive Rate	0.990	0.990	0.980	1.000	0.980	0.970	0.970
	True Negative Rate	0.590	0.630	0.890	0.810	0.890	0.840	0.870
	Accuracy	0.930	0.940	0.970	0.980	0.970	0.960	0.910
	AUC	0.938	0.922	0.899	0.986	0.899	0.873	0.915
Under-sampling	Sensitivity	0.930	0.880	0.910	0.910	0.910	0.920	1.000
	Specificity	0.860	0.860	1.000	1.000	1.000	1.000	0.940
	True Positive Rate	0.980	0.980	1.000	1.000	1.000	1.000	1.000
	True Negative Rate	0.580	0.450	0.570	0.570	0.550	0.600	0.910
	Accuracy	0.920	0.880	0.920	0.920	0.920	0.930	0.970
	AUC	0.893	0.868	0.956	0.956	0.953	0.962	0.968
Over + Under sampling	Sensitivity	0.900	0.950	0.990	0.960	0.970	0.970	1.000
	Specificity	1.000	1.000	0.810	1.000	0.950	1.000	0.950
	True Positive Rate	1.000	1.000	0.980	1.000	0.990	1.000	1.000
	True Negative Rate	0.540	0.680	0.750	0.750	0.770	0.780	0.950
	Accuracy	0.910	0.950	0.970	0.970	0.970	0.970	0.980
	AUC	0.951	0.973	0.899	0.981	0.860	0.984	0.946
SMOTE	Sensitivity	0.960	0.940	0.980	0.980	0.970	0.970	0.970
	Specificity	0.670	0.090	0.710	0.900	0.810	0.860	0.990
	True Positive Rate	0.900	0.990	0.970	0.990	0.980	0.980	0.990
	True Negative Rate	0.670	0.630	0.790	0.830	0.770	0.780	0.990
	Accuracy	0.930	0.940	0.950	0.970	0.960	0.960	0.970
	AUC	0.814	0.856	0.846	0.941	0.891	0.915	0.986

Table 3 reveals that the seven classifiers consistently display high values in the true positive rate, often nearing or achieving 1. Such performance suggests their proficiency in accurately classifying enterprises with good creditworthiness. However, there is evident variance in the true negative rate among classifiers, with metrics like sensitivity and specificity also showing disparities. Intriguingly, different outcomes manifest when using the same classifier but changing the imbalanced sample handling technique. Both the over-sampling and under-sampling methods render suboptimal results for our dataset, particularly in the true negative rates. Similarly, the combined over-and-under sampling method only sometimes fares well across classifiers. In contrast, the SMOTE method shines in sensitivity, specificity, and accuracy. Its substantial improvement in the true negative rate accentuates its effectiveness in rectifying data imbalances, marking it as a favorable approach.

In summary, the XGBoost model showcases consistent classification performance regardless of the applied data balancing techniques, be it random over-sampling, combined over-and-under sampling, or the SMOTE method. XGBoost distinguishes itself from the pack. Its sensitivity, ranging from 96% to 100%, demonstrates its ability to correctly identify between 96% and 100% of creditworthy small and micro enterprises. Regarding specificity, values oscillate between 94% and 100%, reflecting its capability to discern almost all enterprises with credit challenges. All four balanced models boast an accuracy rate that exceeds 91%. In terms of true positive and negative rates, the outcomes are remarkable: 97%–100% of enterprises deemed creditworthy validate this label, while 87%–99% of those marked as less creditworthy genuinely align with this assessment.

**Fig. 3:**
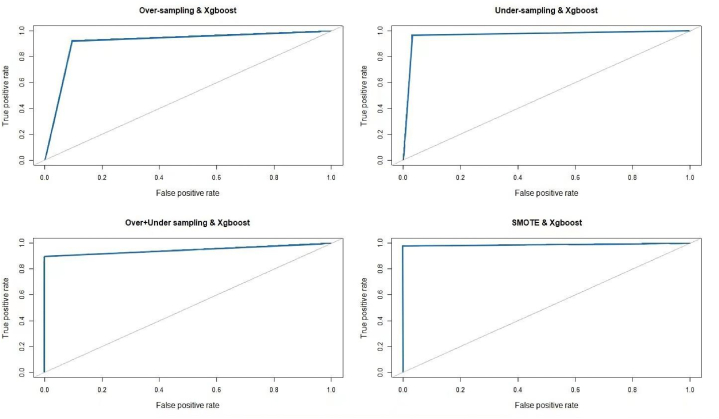
Comparison of ROC curves. Note: The blue line in the figure depicts the ROC curve, and the gray line signifies a Random guess. (For interpretation of the references to colour in this figure legend, the reader is referred to the Web version of this article.)

Notably, the SMOTE-balanced XGBoost model achieves an impressive true negative rate of 99%, underscoring its precision in identifying enterprises prone to default. This enhancement is particularly striking when juxtaposed with results from the other three balancing approaches and deserves special mention. In tandem, the ROC curve and the AUC value thoroughly evaluate the XGBoost model's classification acumen across all balancing methods. A ROC curve gravitating towards the top-left corner, complemented by a superior AUC value, indicates exemplary classification performance. Fig. 3 showcases the ROC curves of XGBoost post-processing via different balancing strategies. Evidently, after balancing with SMOTE, the XGBoost classifier achieves an AUC value of 0.986, with the ROC curve approaching the top-left corner.

### 3.3 Analysis of key nodes

**Fig. 4:**
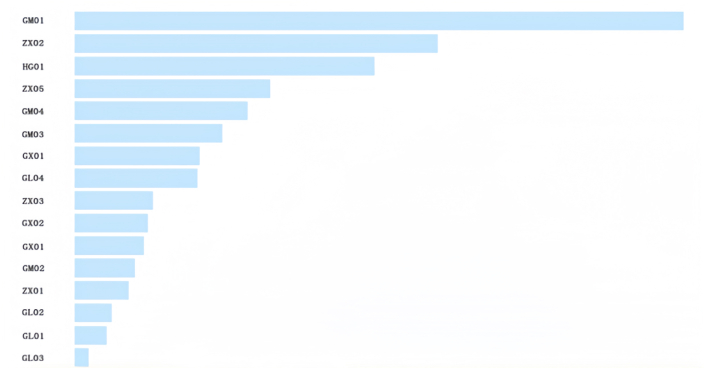
Variable importance ranking.

This research delved into the critical determinants affecting creditworthiness in small and micro enterprises, using the dataset balanced via the SMOTE technique for deeper insights. Leveraging the XGBoost classifier, we discerned and ranked the variables by their significance, as illustrated in Fig. 4. The prominence of the top eight variables markedly surpasses that of the subsequent ones. As a result, this study zeroes in on these eight variables for further analysis: last year's total assets (GM01), return on assets (ROA) of last year (ZX02), compliance performance (HG01), years in operation (ZX05), last year's operating revenue (GM04), last year's total profit (GM03), number of social security contributions/number of employees (GX01), and legal representative's age (GL04).

### 3.4 XGBoost scorecard model

Expanding upon the findings from the previous section, we have selected the top eight variables as predictors, while the creditworthiness of small and micro enterprises acts as the response variable. For our predictive modeling, we employ four renowned machine learning algorithms known for their proficiency in credit assessment: XGBoost, Decision Tree, Random Forest, and SVM [47]. Each algorithm is fine-tuned using Bayesian optimization, working on the same feature subset. 75% of the original dataset, selected randomly, is designated for training the models, with the SMOTE technique applied to address data imbalance. The remaining 25% serves as the test set on which the performance of these models is evaluated.

In our pursuit of a comprehensive evaluation of the model's predictive capabilities, we focus on three key performance metrics: accuracy, F1 score, and AUC. While accuracy generally measures the model's classification prowess across all samples, it is not always adequate, especially when addressing imbalanced datasets. Given the heightened importance of accurately identifying enterprises with low creditworthiness, we supplement accuracy with the F1 score and AUC for a more rounded assessment. The AUC stands as a benchmark metric reflecting a model's quality. It gauges the likelihood that the model will rank a randomly chosen positive instance higher than a negative one. The F1 score, representing the harmonic mean of precision and recall, emphasizes the model's aptitude in distinguishing entities with low creditworthiness. Here, precision speaks to the model's accuracy in positive predictions, whereas recall measures the fraction of actual positives correctly identified. Guided by these vital metrics, we evaluate the performance of our four model variants using the test set, with the outcomes presented in Table 4.

**Table 4:** Predictive results of four types of models.

Model	Accuracy	F1 Score	AUC
XGBoost	99.55%	99.11%	99.08%
Decision Tree	94.09%	96.72%	96.73%
Random Forest	95.57%	97.57%	95.03%
SVM	96.06%	97.84%	97.81%

In Table 4, XGBoost stands out from the rest. It achieves an accuracy surpassing the Decision Tree, Random Forest, and SVM models by 3.46%, 1.98%, and 1.49%, respectively. The distinction is even more pronounced when observing the F1 score, where XGBoost boasts an impressive 99.11%. Regarding AUC, XGBoost only slightly edges out the SVM but has a significant lead over both the Decision Tree and Random Forest models. When using pivotal features, XGBoost consistently demonstrates superior classification ability compared to other top-tier classifiers. Furthermore, its post-feature selection performance considerably eclipses the results prior to feature refinement, highlighting the efficacy of such approaches in evaluating and predicting credit risks among small and micro enterprises, with significant practical implications.

**Fig. 5:**
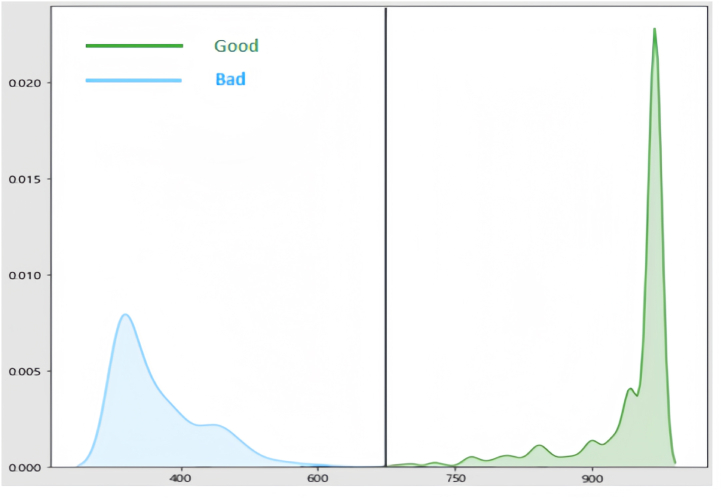
Distribution of scores in the validation dataset.

Further, we construct a credit scoring card based on the XGBoost model. Analyzing the score distribution in the card-creation data, we identify a threshold on the score spectrum. This threshold is carefully adjusted to match business needs, considering factors like approval rate and the overall percentage of bad samples. Results from the validation dataset are illustrated in Fig. 5, revealing that setting a score threshold at 650 points effectively separates most samples. This achieves a remarkable approval rate of 98% while maintaining a low misclassification rate—indicating outstanding overall performance.

Utilizing the credit scoring model developed, we compute credit scores for all 811 enterprises, as detailed in Table 5. The data showcases a significant proportion of small and micro enterprises with impressive credit scores. For instance, those achieving scores of 800 or above make up 68.11% of the dataset. Their high scores categorize them as “excellent” or “good,” indicating their substantial credibility. For such small and micro enterprises, it is advisable to moderately ease the thresholds for financial services, thereby enhancing financing efficiency. For example, commercial banks could establish expedited ‘green channels' for businesses with high credit ratings based on model scoring results. This approach could involve reducing loan interest rates and streamlining the loan application process, making it more efficient and accessible for these enterprises. Conversely, enterprises labeled as ‘bad’ due to poor credit scores constitute only 4.36% of the total. These small and micro enterprises warrant special attention. On one hand, there is a need for credit education to foster awareness and improvement. On the other hand, it is crucial to implement credit disciplinary measures. This includes setting specific thresholds to limit the range of services they can access, especially regarding financial financing. Such measures are essential to mitigate risks and encourage responsible financial behavior. A noteworthy 6.04% of the dataset comprises small and micro enterprises, scoring between 600 and 699. Despite their smaller representation, these businesses should be noticed. Specifically, enterprises with scores ranging from 650 to 699 might be classified as ‘poor,’ yet they are less likely to default than those scoring between 600 and 650. Therefore, dynamic regulatory measures tailored for these specific score ranges could be beneficial. Implementing such nuanced and adaptive regulations can help preserve financial stability while fostering growth for these enterprises.

**Table 5:** Summary of credit ratings for small and micro enterprises.

Credit Score Range	Credit Rating	Percentage of Enterprises	Number of Enterprises with Poor Creditworthiness/Total Enterprises
300–599	bad	4.36%	4.36%
600–699	poor	6.04%	4.17%
700–799	fair	21.49%	1.83%
800–899	good	30.35%	0%
900 and above	excellent	37.76%	0%

## 4 Discussions, limitations and conclusions

### 4.1 Discussions

In collaboration with a third-party enterprise, our team secured a distinctive dataset encompassing financial data, micro-level enterprise behavioral information, public credit records, and third-party sourced information. This comprehensive dataset enabled a multifaceted analysis of credit risk assessment for small and micro enterprises. Nevertheless, our study reveals that not all data elements are equally significant in assessing credit risk. Notably, half of the eight essential variables identified in our model—such as last year's total assets (GM01), return on assets (ROA) of last year (ZX02), compliance performance (HG01), years in operation (ZX05), last year's operating revenue (GM04), last year's total profit (GM03), number of social security contributions/number of employees (GX01), and legal representative's age (GL04)—pertain to financial information. These critical elements underscore that financial information is paramount in evaluating the credit risk of small and micro enterprises, while other non-financial elements also contribute valuable insights.

Identifying critical information elements and choosing the appropriate statistical model are fundamentally linked processes. In a novel approach, we crafted a combination strategy that employed imbalanced algorithms to equalize the original dataset, followed by applying machine learning models to identify significant factors. Our findings revealed that the amalgamation of the SMOTE algorithm with the XGBoost model yielded the most effective classification results, thus establishing a technical baseline for accurately identifying critical factors. Utilizing these key factors, we developed a scoring card model. The research indicated that the feature-optimized XGBoost scoring card model outperformed conventional models like decision trees, random forests, and SVMs. Notably, 68.11% of small and micro enterprises scored above 800, signifying high credibility, whereas 4.36% scored below 600, reflecting poor credibility. This data further illustrates the generally positive credit environment of current small and micro enterprises. Additionally, we proposed dynamic credit regulation strategies tailored to various credit score ranges for the consideration of relevant authorities.

In this study, our findings align with previous research [[4], [5], [6], [7], [8], [9]], indicating that financial information elements are crucial in assessing the credit risk of small and micro enterprises. However, our analysis also suggests that under a multi-information evaluation lens, non-financial elements like enterprise micro-behavioral information, public credit information, and third-party acquired data are significant supplementary dimensions [12], some of which emerge as key factors in our results. Therefore, future research in this area should adopt a holistic approach, considering financial and non-financial elements.

Regarding methodology, while some scholars have successfully applied single machine learning models to predict credit risk in small and micro enterprises [46,47], our study expands on this by integrating imbalanced algorithms with various machine learning models. This approach enables a more profound exploration of high-dimensional information elements and offers a more nuanced method of assessing credit risk in these enterprises. We constructed a credit scoring model using various mainstream machine learning algorithms, evaluated the predictive accuracy among these models, and selected the most effective one for predicting the credit scores of small and micro enterprises, thus overcoming the limitations inherent in using a single model.

Additionally, our research offers practical suggestions to address gaps in the current literature. We found that the majority of small and micro enterprises have high credit scores, with 68.11% scoring above 800, denoting strong credibility. These enterprises could potentially benefit from relaxed financial service requirements, enhancing their access to funding. Conversely, enterprises with very poor credit, constituting 4.36% of our study, demand greater focus, necessitating credit education, disciplinary actions, and setting thresholds to limit their service access, particularly in financial financing. For enterprises scoring between 600 and 699, we advocate against a uniform approach. Instead, a dynamic adjustment mechanism and innovative regulatory classifications are recommended, which can also support the growth of these enterprises.

### 4.2 Limitations

There are still research limitations and room for improvement in this paper. Future research can be considered from the following aspects to improve. Firstly, due to data accessibility, the dataset obtained in this study encompasses only 20 variables, which is relatively limited. The information tags related to small and micro enterprises are increasing in the internet era. Future research can further enrich this, particularly concerning micro-level enterprise behavioral information and public credit information. Secondly, in identifying key factors, this study utilizes a combination of the SMOTE algorithm and the XGBoost model to extract important feature nodes. However, reliance on a single machine learning model may have its limitations. Existing research shows that many scholars are focused on innovation within machine learning models [47]. In future research, we could consider combining imbalanced algorithms with various hybrid machine learning models to uncover significant factors more effectively. Certainly, this approach can also be applied to other fields, such as engineering and management studies. Thirdly, according to the results of the scoring card model, small and micro enterprises with credit scores ranging from 600 to 699 are classified as ‘poor.’ However, there are characteristic differences within this score range among these enterprises, which presents an opportunity for further data mining. For instance, these enterprises can be categorized into different groups, and based on these classifications, tailored credit repair services and financial services can be provided to address their specific needs and challenges.

### 4.3 Conclusions

The primary objective of this study is to enhance the credit risk assessment process for small and micro enterprises, which is an innovative practice in the field. To achieve this, we collected a comprehensive dataset encompassing various dimensions such as financial information, enterprise micro-behavioral information, public credit information, and third-party access to information. We utilized imbalanced sample processing techniques to achieve a more equitable representation of minority-class samples. Subsequently, a machine learning classifier was employed to discern critical factors that adversely affect the credibility of these enterprises. We developed an XGBoost scoring card model based on these identified factors to provide a more nuanced and accurate credit risk assessment for small and micro enterprises. This study draws three main conclusions. Firstly, the integration of the SMOTE algorithm with the XGBoost model exhibits certain performance advantages in handling imbalanced datasets; secondly, trustworthy financial information remains at the heart of crucial risk determinants; thirdly, the XGBoost scoring card model based on significant features effectively enhances the accuracy of credit risk assessment. These insights provide theoretical contributions and practical implications for credit risk assessment of small and micro enterprises.

(1) Theoretical contributions

A prevalent challenge in the credit risk assessment of small and micro enterprises is the imbalance in the number of ‘default’ versus ‘non-default’ entities. Our study addresses this issue by employing a diverse array of imbalance treatment methods, including Over-sampling, Under-sampling, Combined Over and Under-sampling, and SMOTE, which were integrated with various machine learning algorithms such as CART, C4.5, RF, SVM, AdaBoost, Bagging, and XGBoost. Notably, the synergy between the SMOTE algorithm and the XGBoost model slightly outperformed other combinations across multiple evaluation metrics. This approach not only mitigates the issue of underrepresented minority class samples but also brings forth a novel perspective in the theoretical exploration and practical application of imbalanced algorithms.

In tandem with imbalanced algorithms, our research leveraged established machine learning models for feature dimensionality reduction. This method proved effective in identifying critical factors influencing the credit risk assessment in small and micro enterprises. The strategic application of machine learning models for feature reduction successfully processes high-dimensional data, thus facilitating a reduction in data volume and computational complexity. It also plays a crucial role in circumventing potential issues like overfitting. The key feature nodes unearthed through machine learning models have substantial implications, offering valuable insights for both theoretical and practical aspects of credit risk assessment in this sector.

Furthermore, our research introduces the XGBoost scoring card model, grounded on pivotal feature nodes, which surpasses conventional machine learning models in terms of predictive accuracy. This finding makes a significant theoretical contribution to the methodological domain of the model. It underscores that mere innovation in model ensembles does not necessarily translate to improved predictive accuracy. Instead, constructing a robust machine learning model, firmly based on critical features and underpinned by a solid theoretical foundation, can lead to enhanced predictive results. This insight opens new avenues for future research in model development and application within the realm of small and micro enterprise credit risk assessments.

(2) Practical implications

Our study found that half of the eight key variables identified are linked to financial data, such as last year's total assets, return on assets (ROA) of last year, last year's operating revenue, and last year's total profit. Furthermore, non-financial indicators, including compliance performance (a public credit indicator), years in operation, and legal representative's age (micro-behavioral information), demonstrate correlations with the credit risk of small and micro enterprises. Assessors, whether conducting subjective or objective evaluations, should give these elements, particularly the financial aspects, significant consideration. Our findings also reveal that non-financial data, like micro-behavioral information, public credit records, and third-party information, serve as essential supplementary factors in a multi-dimensional evaluation framework.

A substantial segment of small and micro enterprises in our study boasts high credit scores, with 68.11% scoring over 800, indicative of strong credibility. These businesses could gain from relaxed criteria in financial services, potentially improving their access to funding. In contrast, enterprises with markedly low credit scores, which comprise 4.36% of our sample, warrant additional focus. Such entities require in-depth credit education, strict disciplinary measures, and defined thresholds for service accessibility, especially in financial financing. For those with credit scores between 600 and 699, a generalized approach proves ineffective. Instead, we advocate for dynamic adjustment mechanisms and novel regulatory frameworks to support the development of these enterprises. Our approach to credit risk assessment and management offers valuable insights for practitioners and policymakers in the field, aiming to enhance financial stability and growth among small and micro enterprises.

## Funding

This research was financially supported by the Ministry of Education of the People's Republic of China Humanities and Social Sciences Youth Foundation (Grant numbers: 22YJCZH038), Zhejiang Province Statistical Research Project (Grant numbers: 23TJQN18), Zhejiang Province Department of Education (Grant numbers: Y202351047), and Humanities and Social Science Fund of Ministry of Education of the People's Republic of China (Grant numbers: 23YJA840015).

## Data availability statement

Data will be made available upon request and can be accessed by contacting the corresponding author.

## CRediT authorship contribution statement

**Zhouyi Gu:** Writing – original draft, Methodology, Funding acquisition, Formal analysis. **Jiayan Lv:** Software, Methodology. **Bingya Wu:** Writing – review & editing, Supervision, Data curation. **Zhihui Hu:** Methodology. **Xinwei Yu:** Software.

## Declaration of competing interest

The authors declare that they have no known competing financial interests or personal relationships that could have appeared to influence the work reported in this paper.

## Footnotes

1 See https://www.worldbank.org/en/topic/smefinance.

2 See http://www.china.org.cn/china/2019-09/22/content_75232933_2.htm for more details.
